# Prior medication adherence of participants and non participants of a randomized controlled trial to improve patient adherence in cardiovascular risk management

**DOI:** 10.1186/s12874-019-0743-7

**Published:** 2019-05-09

**Authors:** A. Sieben, S. J. H. Bredie, J. C. H. B. M. Luijten, C. J. H. M. van Laarhoven, S. van Dulmen, H. A. W. van Onzenoort

**Affiliations:** 1 Department of Surgery, Division of Vascular Surgery, Radboud university medical center, Geert Grooteplein 10, Postbus 9101, 6500 HB Nijmegen, the Netherlands; 2 Department of General Internal Medicine, Division of Vascular Medicine, Radboud university medical center, Nijmegen, the Netherlands; 3 Department of General Surgery, Radboud university medical center, Nijmegen, the Netherlands; 4 Department of Primary and Community Care, Radboud university medical center, Radboud Institute for Health Sciences, Nijmegen, the Netherlands; 5 NIVEL (Netherlands institute for health services research), Utrecht, the Netherlands; 6 Faculty of Health and Social Sciences, University of South-Eastern Norway, Drammen, Norway; 7 Department of Clinical Pharmacy, Amphia Hospital, Breda, the Netherlands; 8 Department of Clinical Pharmacy and Toxicology, Maastricht University Medical Center+, Maastricht, the Netherlands

**Keywords:** Randomized controlled trials, Informed consent, Participation, Selection bias, Adherence

## Abstract

**Background:**

Poor medication adherence is a major factor in the secondary prevention of cardiovascular diseases (CVD) and contributes to increased morbidity, mortality, and costs. Interventions for improving medication adherence may have limited effects as a consequence of self selection of already highly adherent participants into clinical trials.

**Methods:**

In this retrospective cohort study, existing levels of medication adherence were examined in self-decided participants and non-participants prior to inclusion in a randomized controlled study (RCT), evaluating the effect of an intervention to improve adherence. In addition, the non-participants were further divided into ‘responders’ and ‘non responders’. All individuals had manifest cardiovascular disease and completed a questionnaire with baseline characteristics, the Beliefs about Medicines Questionnaire (BMQ) and the Modified Morisky Scale® (MMS®) as part of a regular screening program. A logistic regression was conducted to examine the relationship between study participation willingness, adherence level and the beliefs about medication.

**Results:**

According to the MMS® the adherence level was comparable in all groups. In both (non)-participants groups, 36% was classified as high adherent; 46% participants versus 44% non-participants were classified as medium adherent and 19% of the participants versus 20% of the non-participants were low adherent (*p* = 0.91. The necessity concern differential (NCD) from the BMQ was 3.8 for participants and 3.4 for non-participants (*p* = 0.32).

**Conclusion:**

This study shows that adherence to medication and beliefs about medication do not differ between participants and non-participants before consenting to participate in an RCT. The study design seems not to have led to greater adherence in the study group.

## Background

Cardiovascular risk reduction is predominantly based on lipid and blood pressure lowering treatment, inhibition of platelet aggregation, smoking cessation and control of obesity [1]. A limitation in lowering cardiovascular risk is poor adherence to prescribed medication [2] which may consequently can lead to increased morbidity, mortality and costs [3–6]. Nevertheless, a recent review with mostly cohort studies, showed that only 60% of people who use cardiovascular medication, were adherent to their cardiovascular medication [7]. In view of that there is a need for interventions to improve medication adherence in this population. Although there is a considerable amount of research in the field of interventions to improve medication adherence in cardiovascular patients, they often show only small effects [8]. It is suggested that patient recruitment methods in randomized controlled trials (RCT) to improve patient adherence to medication may influence outcome [8, 9]. An important observation is that patients participating in RCTs generally have higher adherence rates at baseline than could be expected based on observational studies [10–14]. It is conceivable that the informed consent procedure results in a selection of patients with higher adherence rates [12]. Willingness to participate is positively influenced by patients’ engagement with their medical condition, high level of education and the influence of an important person [11, 15]. These characteristics are also considered as positive determinants for medication adherence [16]. Although a recent review showed that the inclusion of non adherent patients was the single feature significantly associated with effective adherence interventions, most studies seem to include patients because they are willing to participate not because they are poor adherent [11]. It is suggested that patients participating in these RCTs already have a pre-existing high adherence level at baseline [10, 12–14]. Selection of participants with high levels of adherence at baseline, makes it difficult to show an improving intervention effect (ceiling effect) [8]. More understanding about the medication adherence of participants as well as non-participants before the start of these RCT’s may contribute to a better understanding of why so many studies show no improvement in medication adherence. One possible explanatory determinant for (non) adherent behaviour is medication beliefs. Personal beliefs about need for treatment (necessity beliefs) and concerns about several potential adverse consequences (concern beliefs) could explain a large part of (non) adherent behaviour [16–18]. If patients perceive that the need for medication outweighs the concerns, they are more likely to be adherent to their medication(s) [19].

## Methods

### Aim

The aim of this study is to explore possible differences in adherence to existing prescribed medication in cardiovascular patients who did or did not consent to participate in an RCT which expressly explored the effects of an intervention to improve adherence. We hypothesized that patients who are willing to participate in a clinical trial are more likely to be medication adherent and have more ‘necessity beliefs’ about their medication compared to patients who are not willing to participate.

### Study design and setting

In this retrospective cohort study we included patients who participated or declined participation in the (MIRROR) trial (a Multifaceted nurse -and web-based Intervention for impRoving adheRence to treatment in patients with cardiOvasculaR disease) [20]. In brief, the MIRROR trial was a prospective, randomized controlled trial in which patients aged ≥18 years and diagnosed with a manifest cardiovascular disease (i.e. acute coronary syndrome, peripheral arterial disease or stroke/Transient Ischemic Attack (TIA)) after providing written informed consent, were included. The MIRROR trial aimed to study the effect of different adherence enhancing strategies on cardiovascular medication adherence. Within this context, patients were randomized to usual care, an e-health intervention, and an e-health intervention combined with motivational technique consultations.

### Participants

All patients referred to the Radboud University Medical Center with a new diagnosis of acute coronary syndrome, myocardial infarction, peripheral arterial disease, an aneurysm of the aorta or TIA or stroke over the prior 6 weeks were included into the hospital CVD screening program. This screening program aims to identify cardiovascular risk factors and consists of screening for lifestyle (smoking, diet and exercise), medication adherence by the self reported questionnaires Modified Morisky Scale® (MMS®) and the Beliefs about Medication Questionnaire (BMQ), blood lipid levels, blood pressure, waist circumference, body mass index (BMI), glucose blood levels and a family history of cardiovascular diseases. If indicated, preventive therapies (medication and lifestyle interventions) are initiated and followed over time [1]. All patients referred to this screening program were asked to participate in the MIRROR- trial. ‘Participants’ were patients who were willing to participate in the intervention study and ‘non-participants’ were patients who declined informed consent for the MIRROR trial. Because adherence to medication may also differ between responders and non- responders to surveys, with responders having higher adherence levels [21] we divided the group of non-participants further. Retrospectively of the MIRROR trial, a letter was sent to all non-participants for a different study not subject to this paper. For this study, a signed informed consent was requested from the non-participants. Non-responders were patients who did not sign this letter. Responders were patients who signed the informed consent letter. We aimed to explore if the non-responding subgroup of the non-participants differed from the responders with respect to their level of medication adherence on the basis of prior MMS® from the screening program.

### Ethical approval

The Ethical Committee waived the need for a formal informed consent for this study. The study was conducted according to the good clinical practice protocol and we used usual care data considering the research question of this study. Data was anonymized according to the research protocols of the Ethical Committee.

### Outcomes

Participation or declining to participate to the RCT was the independent variable in this study. Adherence to medication and the beliefs about medication the dependent variables. Adherence to cardiovascular medication was calculated by the MMS® [22–24]. It consists of eight items aimed at measuring adherence. Each item accounts for 0 or 1 in the case questions are answered by no or yes respectively. Consequently, total MMS® scores range between 0 and 8. These scores were divided into three levels of adherence: low adherence (sum score < 6), medium adherence (sum score 6 to < 8) and high adherence (sum score of 8) [25]. To evaluate patients’ beliefs and perceptions about their medication, BMQ [26] was used. This validated questionnaire provides information about the beliefs, perceived necessity and concerns the patient has regarding their illness and prescribed medication. There are five statements regarding “necessity beliefs” and five regarding “concern beliefs”. Patients indicated their degree of agreement with each individual statement about the use of their medicines on a five-point Likert scale. Thus, total scores for the necessity and concerns scales could range from 5 to 25. The necessity– concerns differential (NCD) was then calculated as the difference between necessity and concerns scores and had a possible range of − 20 to 20 [19, 27]. To differentiate between patients on the basis of their beliefs about the necessity of their medication and their concerns about taking medication, the total necessity and concern scores [5−25] were split at midpoint (thus 5–12 was considered as low and 13 t/m 25 was considered as high). Patients were then classified into four different categories: accepting (high necessity and low concerns), ambivalent (high necessity and high concerns), skeptical (high concerns and low necessity) and indifferent (low concerns and low necessity) [28–30].

From all patients the type of CVD (acute coronary syndrome, myocardial infarction, peripheral arterial disease, an aneurysm of the aorta or TIA) was recorded. Also, the following baseline and clinical characteristics were collected: age, sex, level of education, employment status, the country of origin and the type of cardiovascular medication used.

### Data collection and timeline

Data were derived from the screening program. Data were registered in a secure website which could only be accessed by nurses involved in the screening program. Within, on average, six weeks after the CVD-event, baseline characteristics and the questionnaires were collected for all patients as part of the screening program.

### Statistical analyses

Data were analyzed and evaluated using SPSS version 22. Descriptive statistics (mean, median, standard deviation) were used for all variables. A Mann-Whitney Test was used to compare groups (participants and non-participants) with the non parametric outcome, the MMS®. Confounders were explored by performing a logistic linear test of all the characteristics in the case the groups significantly differed (including the NCD). The same Mann-Whitney test was performed to compare the NCD between the groups. A logistic regression was used to explore differences between (non) participants and the four belief groups. The same statistical analyses were performed for the responders and non-responders. A Kruskal-Wallis test was performed to explore the relationship between the NCD and the MMS® for all groups.

## Results

**Fig. 1:**
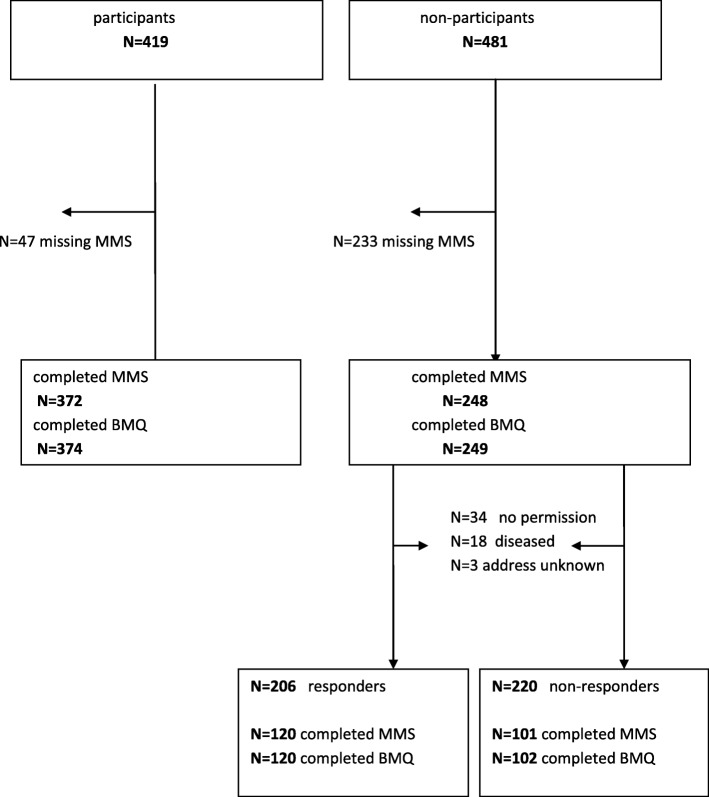
Participants, non-participants,responders and non responders and MMS and BMQ

In total, 900 patients with a new cardiovascular event between October 2011 and October 2013 were eligible for participation into the MIRROR trial. Of these, 419 agreed and 481 refused participation. Of all the non-participants who received a letter for another study, 220 did not respond. Consequently, 261 non-participants were classified as responders (Fig. 1).

### Patient characteristics

The total cohort (participants and non-participants) had a mean age of 62 years and was predominantly male (67%). Participants significantly differed from non-participants with respect to age (61 years versus 63 years, *p* = 0.001), male sex (71% versus 58%, *p* = 0.001), systolic blood pressure (136 mmHg versus 142 mmHg, *p* = 0.001). Participants were more frequently diagnosed with an acute coronary syndrome (36% versus 16%, *p* < 0.001]), were using more beta blocking agents or agents acting on the renin-angiotensin system (58% versus 46% *p* = 0.001 and 59% versus 44% *p* = 0.001, respectively), and were using more lipid lowering medication (94% versus 82%, *p* < 0.001). Among the non-participants, responders were older (64 versus 60 years, *p* = 0.002) and used more agents acting on the renin-angiotensin system (48% versus 37%, *p* = 0.02) than non-responders (Tables 1 and 2).

**Table 1:** Differences in patient characteristics between participants and non participants in a RCT-trial on adherence

	Participants [*n* = 419]	Non-participants [*n* = 481]	*P*-value
Age [mean, ±SD)	60.5 [±10]	63 [11]	0.001
Gender (N [%])			
Male	296 [7]	279 [58]	< 0.001
Female	123 [29	202 [42]	
Education level (N[%])			< 0.001
Primary	66 [18]	141 [31]	
Secondary	185 [49]	180 [39]	
University	124 [33]	140 [30]	
Labour (N[%])			0.08
Paid labour	137 [37]	123 [27]	
Unemployed	98 [26.3]	138 [30]	
Retired	138 [37]	199 [43.3]	
Country of origin is the Netherland (N[%])			0.66
Yes	327 [90]	398 [86]	
No	37 [10]	64 [14]	
Reason referral (N[%])			< 0.001
acute coronary syndrome	150 [36]	79 [16]	
peripheral arterial disease	71 [17]	101 [21]	
troke/TIA	198 [47]	301 [63]	
Blood pressure (mmHg; mean ± SD)			< 0.001
Systolic	136 [±18]	142 [±20]	0.23
Diastolic	77 [±11]	78 [±11]	
Body Mass Index (mean ± SD)	27 [±4]	26 [±4]	0.30
Waist (mean ± SD)			
Male	99.5 [±9]	98.4 [±12]	0.10
Female	92 [±14]	90 [±13]	0.07
Lipids (mmol/ltr, mean SD)			
Totaal cholesterol	4.5 [±1.1]	4.6 [±1]	0.7
Triglyceriden	1.8 [±1]	1.7 [±1]	0.01
HDL	1.2 [±0.3]	1.2 [±0.3]	0.002
LDL	2.5 [±0.9]	2.6 [±0.9]	0.66
Medication (N [%])			
Antithrombotic agents [ATC B01]	404 [98]	461 [98]	0.78
Diuretics [ATC C03]	109 [26]	135 [29]	0.44
Beta Blocking agents [ATC C07]	239 [58]	218 [46]	0.001
Calcium channel blockers [ATCC08]	65 [16]	72 [15]	0.86
Agents acting on [..] system [ATC C09]	244 [59]	206 [44]	0.001
Lipid modifying agents [ATC C10]	387 [94]	384 [82]	< 0.001

**Table 2:** Differences in patient characteristics between responders and non-responders

	responder [*n* = 206]	Non-responder [*n* = 220]	*P*-value
Age (mean ± SD)	64 [10]	60 [12]	0.002
Gender (N [%])			
Male	120 [58]	129 [59]	0.93
Female	86 [42]	91 [41]	
Education level (N[%])			
Primary	52 [26]	67 [32]	0.43
Secondary	80 [41]	81 [39]	
University	66 [33]	62 [29]	
Labour (N [%])			
Paid labour	49 [25]	68 [33]	0.08
Unemployed	55 [28]	64 [31]	
Retired	94 [47]	77 [36]	
Country of origin is the Netherlands(N[%])			
Yes	174 [88]	181 [86]	0.62
No	24 [12]	29 [14]	
Reason referral (N[%])			0.89
acute coronary syndrome	34 [16]	37 [17]	
peripheral arterial disease	47 [23]	46 [21]	
stroke/TIA	125 [61]	137 [62]	
Blood pressure (mmHg; mean ± SD)			
Systolic	140 [±19]	142 [±20]	0.30
Diastolic	78 [±11]	79 [±10]	0.23
Body Mass Index (mean ± SD)	26 [±4]	26 [±4]	0.22
Waist (mean ± SD)			
Male	97 [±11]	99 [±12]	0.16
Female	91 [±13]	89 [±13]	0.36
Lipids (mmol/ltr; mean ± SD)			
Totaal cholesterol	4.6 [±1]	4.6 [±0.9]	0.73
Triglyceriden	1.7 [±1]	1.7 [±0.9]	0.01
HDL	1.3 [±0.3]	1.2 [±0.3]	0.06
LDL	2.5 [±0.9]	2.6 [±0.9]	0.78
Medication(N [%])			
Antithrombotic agents [ATC B01]	196 [97]	213 [98]	0.27
Diuretics [ATC C03]	62 [31]	56 [26]	0.28
Beta Blocking agents [ATC C07]	93 [46]	96 [44]	0.74
Calcium channel blockers [ATCC08]	30 [15]	34 [16]	0.80
Agents acting on [..] system [ATC C09]	98 [48]	80 [37]	0.02
Lipid modifying agents [ATC C10]	166 [82]	173 [80]	0.59

### Medication adherence

We did not observe differences in adherence measured by the MMS® between both groups (*p* = 0.99). According to the MMS® 19% of the participants was classified as low adherers compared to 20% in the non-participants group. Forty-six percent of the participants and 44% of the non-participants were classified as medium adherers, whereas 36 and 37% were classified as high adherers, respectively. There were no differences in adherence according to the MMS® between responders and non-responders (*p* = 0.47). In both the responders and non-responders group, 36% scored high on adherence. Sixteen percent of the responders were low adherent compared to 24% in the non-responder group. Forty-eight percent of the responder group scored a medium adherence and 41% of the non-responders. Compared to study participation all characteristics that significantly differed between both groups were separately analyzed by a logistic regression analyses. None of the variables significantly influenced the association between study participation and adherence according to the MMS® (Tables 3 and 4).

**Table 3:** Differences participants and non-participants in adherence and beliefs about medication

	Totaal	Non-participants	Participants	P-value
Adherence according to the MMS N [%]				0.99
Low adherence	119 [19]	49 [20]	70 [19]	
Medium adherence	279 [45]	109 [44]	170 [46]	
High adherence	222 [36]	90 [36]	132 [35]	
NCD mean [SD]	3.65[±4.8]	3.4 [±5]	3.8 [±4.9]	0.13
Belief Groups [N%]				0.23
Accepting	160 [26]	61 [24]	100 [27]	
Ambivalent	418 [67]	165 [67]	255 [68]	
Sceptical	19 [3]	10 [4]	9 [2]	
Indifferent	23 [4]	13 [10]	10 [3]

**Table 4:** Differences responders and non-responders in adherence and belief about medication

	Totaal	Non-responders	Responders	*P*-value
Adherence according to the MMS N [%]				0.47
Low adherence	43 [20]	24 [24]	19 [16]	
Medium adherence	99 [45]	41 [40]	58 [48]	
High adherence	79 [36]	36 [36]	43 [36]	
NCD mean [SD]	3.6 [±4.9]	3.1 [±5]	4 [±4.9]	0.17
Belief Groups [N%]				0.001
Accepting	56 [25]	24 [24]	32 [27]	
Ambivalent	148 [67]	62 [61]	86 [72]	
Sceptical	8 [4]	6 [6]	2 [2]	
Indifferent	10 [4]	10 [9]	0 [0]	

### Beliefs about medication

Based on the BMQ the necessity concerns differential (NCD) was 3.8 among participants compared to 3.4 among non-participants (*p* = 0.13). Of all the participating and non-participating patients 26% were in the accepting group, 67% in the ambivalent group, 3% in the skeptical and 4% in the indifferent group. No differences were observed between the two groups (*p* = 0.23). The mean score of the NCD in the responders and non-responders groups was 3.6 and 3.1 respectively (*p* = 0.21). Among the non-responders 24% were in the accepting group, 61% in the ambivalent group, 6% in the skeptical group and 9% in the indifferent group. For the responders this was 27, 72, 2 and 0% respectively. Differences between both groups were statistically significant (*p* < 0.01). Logistic regression analysis on NCD did not significantly influence the association between study participation and adherence according to the MMS®.

## Discussion

To our knowledge this is the first study exploring the differences in medication adherence in patients who did or did not consent to participate in an RCT evaluating the effect of an intervention to improve medication adherence. Our study showed that patients willing to participate in an RCT evaluating the effect of an intervention to improve medication adherence, have a comparable adherence level to patients who declined participation. Even by further exploring the non-participant group in responders and non-responders, we did not observe differences in adherence between the groups. Consequently, the results of this study suggest that a population representative in adherence level participated in an RCT evaluating the effect of an intervention to improve medication adherence.

Previous studies suggested that patients not participating in RCTs to improve medication adherence have a different pre-existing adherence level from patients who participate [10–14]. This was supported by the observed differences in adherence levels between these RCTs and observational studies [10–14]. Typically, adherence levels among patients in RCTs were higher than in observational studies. Although not different among participants and non-participants, adherence in this study was also high. An explanation for the high adherence rate in both groups could be that we started inclusion for the RCT within six weeks after the cardiovascular event. For cardiovascular patients who just had an event, the need for adherent behaviour is emerging [31, 32]. Yet, as the event fades and symptoms subside, adherence levels can also decline [33]. Research with a long follow up is needed to establish if there will be a difference in adherence between participants and non-participants over time.

In all groups, we observed significant differences in patient characteristics. Compared to non-participants, participants were younger and more were highly educated. This was also observed among responders and non-responders. These are known as prognostic characteristics for patients who are willing to participate in a clinical trial [15] and for a high adherence level [34, 35]. Although the relationship between socio-demographic variables and adherence is mainly weak and inconsistent [34, 36, 37] it was expected that these characteristics could have been an explanation for the assumed higher adherence rates in the participant groups. However, we could not support this hypothesis. Also, next to the high adherence rate, a high mean NCD score was present in all groups. This only confirmed the adherent behaviour in both groups [19]. It is also congruent with earlier studies showing that medication beliefs can be a more powerful predictor of medication adherence than clinical and socio-demographic factors [19, 38]. However, we did not observe a relationship between NCD and trial participation. We did observe a significant difference in the beliefs about medication groups in the (non) responder groups. More patients of the non-responder group were also in the indifferent and skeptical group. Non-responders of surveys are known for more negative evaluations of healthcare [39]. This could be associated with higher concern beliefs in medication as these are partly influenced by the prescriber-patient relationship in healthcare [40]. The number of patients who differed in these groups was very small and the NCD did not differ. More research is needed to draw any conclusions on this point.

This study had some limitations. We had to deal with missing data especially in the self reported questionnaires BMQ and MMS®. There were fewer missing in the participants group compared to the non-participants group. The questionnaires were just implemented in the screening program. As the questionnaires were also part of the MIRROR trial, more attention could have been paid to participants for documenting these questionnaires. So there were more patients in the non-participant group who did not fill out the MMS®. These patients could very well be non-adherent [21]. There are different methods available to measure adherence. Each method has advantages and disadvantages. The MMS® [22, 24, 41] is a validated questionnaire that can be easily applied to large populations. As MMS® is a subjective measure, adherence levels may be higher than what is expected in real life. Refill data from the out-patient pharmacy on the other hand has been used extensively to provide insight into drug acquisition and dispensing [42]. However, to use the pharmacy refill data we need an informed consent from patients. This study however used data from patients collected only in standard care because we wanted to include patients who declined participation in a RCT. Other methods, such as MEMS or pill count, seem to influence patient’s behavior through direct confrontation. Moreover, application of MEMS is relatively expensive, especially when applied in standard care [42]. The BMQ was used because, to our knowledge, is the only validated questionnaire that evaluates patients’ beliefs, necessity and concerns the patient has according to his illness and prescribed medication. This in contrast to other validated adherence questionnaires that measure specific medication-taking behavior of patients [26, 43]. The high NCD score confirmed the prediction of adherent behaviour in both groups [19, 38] .

## Conclusion

This study showed no differences in medication adherence between participants and non-participants prior to the inclusion of the MIRROR trial. A representative group seems to have participated in this randomized controlled trial designed to improve medication adherence [20].

## Abbreviations

BMI: Body mass index
BMQ: Beliefs about Medicines Questionnaire
CVD: Cardiovascular diseases
MIRROR trial: A Multifaceted nurse -and web-based Intervention for impRoving adheRence to treatment in patients with cardiOvasculaR disease
MMS®: Modified Morisky Scale®
NCD: Necessity concern differential
RCT: Randomized controlled study
TIA: Transient Ischemic Attack
